# Spontaneous pregnancies in patients with at least one failed IVF cycle after the management of autoimmune disorders, hereditary thrombophilia, and methylation disorders

**DOI:** 10.5935/1518-0557.20190034

**Published:** 2019

**Authors:** Atakan Tanacan, Mehmet Sinan Beksac

**Affiliations:** 1 Division of Perinatology, Department of Obstetrics and Gynecology, Hacettepe University, Ankara, Turkey

**Keywords:** infertility, *in vitro* fertilization, methylenetetrahydrofolate reductase, autoimmunity

## Abstract

**Objective:**

This study aimed to describe the impact on achieving spontaneous pregnancy of treating patients with at least one failed in-vitro fertilization (IVF) cycle for autoimmune disorders, hereditary thrombophilia, and methylation disorders.

**Methods:**

Fifty-three patients who met the enrollment criteria seen between January 2007 and October 2017 were included in this retrospective cohort study. The patients were retrospectively assessed for the presence of hereditary thrombophilia, methylenetetrahydrofolate reductase (MTHFR) polymorphisms, serum vitamin B12/folate/homocysteine levels, and autoimmune antibody positivity. The required data were extracted from the institutional patient database. Statistical analyses were performed on Statistical Package for the Social Sciences (SPSS.22^®^). The Kolmogorov-Smirnov test was used to evaluate the distribution of the data, and since the data did not following a normal distribution, proportions and median (minimum-maximum) values were used.

**Results:**

The 53 patients included in the study had singleton pregnancies. The distribution of autoantibodies was as follows: thyroid peroxidase (n=17); antithyroglobulin (n=11); double-stranded DNA (n=4); antinuclear (n=8); anti-smooth muscle (n=1); and anticardiolipin IgG and IgM (n=1). Autoimmune diseases included Hashimoto's thyroiditis (n=23); SLE (n=7); Behcet's disease (n=1); Sjogren's syndrome (n=1); ulcerative colitis (n=1); and anti-phospholipid antibody syndrome (n=1). Ten patients had heterozygous Factor V Leiden thrombophilia; two had homozygous Factor 5 Leiden thrombophilia; and three had the prothrombin 20210A heterozygous mutation. Twenty-eight patients were positive for autoantibodies and hereditary thrombophilia and/or MTHFR polymorphisms.

**Conclusions:**

Evaluation and management of hereditary thrombophilia, MTHFR gene polymorphisms, and/or autoimmune conditions may be beneficial for patients with unexplained infertility.

## INTRODUCTION

Infertility is defined as a couple's inability to achieve pregnancy after abstaining from contraceptive methods for 12 months. The prevalence of primary infertility - a term coined to categorize couples unable to achieve their first pregnancy - is approximately 1.9% (Mascarenhas *et al* ., 2012). The time interval accepted for women aged 35 years or older is six months (Practice Committee of American Society for Reproductive Medicine, 2013).

In the absence of an explainable cause after comprehensive examination, couples are diagnosed with unexplained infertility (UI). Comprehensive examination is expected to reveal the following: 1) regular ovulation, 2) tubal patency, 3) a normal uterine cavity, 4) normal semen analysis results, and 5) an adequate ovarian oocyte reserve (Practice Committee of American Society for Reproductive Medicine, 2013). Expectant management, lifestyle changes, timed intercourse, and intrauterine insemination (IUI) with gonadotropin stimulation or *in vitro* fertilization (IVF) are different treatment options for UI (Nandi *et al* ., 2017; Quaas & Dokras, 2008).

IVF has been an effective treatment method for infertile couples performed with high success rates for nearly four decades (Steptoe & Edwards, 1978; Elizur *et al*., 2006). It has become the preferred treatment modality for individuals with tubal factor infertility, diminished ovarian reserve, and severe male factor infertility (Rongieres, 2015). However, the role of IVF in patients with UI has not been clearly determined (Rongieres, 2015).

Maternal health disorders (epigenetic, metabolic, immunological conditions, etc.) that affect gametogenesis, fertilization, and implantation may also be linked to UI (Azem *et al*., 2004; Deroux *et al*., 2017; Coulam & Jeyendran, 2009; Zhong *et al*., 2012; Qublan *et al*., 2006). It has been reported that metabolic pathway disorders including aminoacyl-tRNA biosynthesis, "glycine-serine-threonine" metabolism, "alanine-aspartate-glutamate" metabolism, "valine-leucine-isoleucine" biosynthesis, and methionine metabolism may be linked to impaired gametogenesis and/or unsuccessful implantation (Spiridonov *et al*., 2005; Ni *et al*., 2016; Unni *et al*., 2015). Autoimmune disorders (autoimmune antibody positivity and related diseases) may also be responsible for undesired biological processes such as uterine inflammation, which further lead to impaired endometrial receptivity and placentation (Deroux *et al*., 2017; Chen *et al* ., 2017). Thus, methylation disorders and immunological conditions seem to play important roles in the pathophysiology of UI (Azem *et al*., 2004; Coulam & Jeyendran, 2009).

This study aimed to describe the impact on achieving spontaneous pregnancy of treating patients with at least one failed in-vitro fertilization (IVF) cycle for autoimmune disorders, hereditary thrombophilia, and methylation disorders. 

## MATERIALS AND METHODS

We evaluated 53 patients with at least one failed IVF cycle for the treatment of primary UI who became pregnant spontaneously and gave birth at our hospital (between January 2007-October 2017) as a consequence of proper management of their medical conditions (autoimmune disorders, hereditary thrombophilia, and MTHFR polymorphisms). The required data were extracted from the Hacettepe University patient database.

All patients with primary UI and at least one failed IVF cycle were included in a special follow-up program and evaluated before conception. The patients were screened for hereditary thrombophilias (factor V Leiden mutation, prothrombin 20210A mutation, protein C/S deficiency), MTHFR polymorphisms, serum vitamin B12/folate/homocysteine levels, autoimmune antibody positivity (antinuclear (ANA), thyroid peroxidase (TPO), antithyroglobulin (TgAb), anti-parietal cell (APA), and antiphospholipid antibodies depending on their clinical findings and family history), and related disease/syndromes at the Hacettepe University, Division of Perinatology. Patients in a quiescent period in terms of immunological and metabolic inflammatory processes were allowed to get pregnant.

All patients were included in a special antenatal care program and had low molecular weight heparin (LMWH = enoxaparin 2000 Anti-Xa IU/0.2 ml), oral prednisone (methylprednisolone 4 mg), and aspirin (acetylsalicylic acid 100 mg) added to their specific treatment protocols as soon as they became pregnant. A special low-methionine diet was prescribed to patients with MTHFR polymorphisms.

Pregnancy follow-up comprised serial laboratory testing and ultrasound examination, aneuploidy screening (combined or triple test), fetal anatomy scanning at the 20^th^-24^th^ week of gestation, oral glucose challenge test, and non-stress tests weekly (after the 28^th^ week of gestation). All medications were stopped three days before delivery.

The median (minimum-maximum) values of patient age; duration of infertility; number of previous IVF cycles; serum folate, vitamin B12, and homocysteine levels just before conception; gestational week at birth; birth weight; and 5-minute APGAR scores were calculated, as the data did not follow a normal distribution. The proportions of patients with positive tests for autoantibodies, autoimmune diseases, hereditary thrombophilia, and MTHFR polymorphisms were also recorded.

Statistical analyses were performed on the Statistical Package for the Social Sciences (SPSS.22^®^, IBM SPSS Statistics for Windows, Version 22.0. Armonk, NY: IBM Corp.). The Kolmogorov-Smirnov test was used to assess the distribution of the data, and since the data did not follow a normal distribution, proportions and median (minimum-maximum) values were used.

This study was approved by the Ethics Committe at Hacettepe University Et (GO 18/158).

## RESULTS

The 53 singleton pregnancies resulted in live births. There were no ectopic pregnancies, miscarriages, or stillbirths. There were only three spontaneous late preterm births (5.6%) (at 34, 35, and 35 weeks of gestation). Preeclampsia, eclampsia, and placental abruption were not observed in any of the patients.

Table 1 shows the median and minimum-maximum values for maternal age; number of previous IVF cycles; duration of infertility; gestational week at birth; birth weight; 5-minute APGAR score; and serum folate, vitamin B12, and homocysteine levels.

**Table 1:** Descriptive statistics for maternal age; number of previous IVF cycles; duration of infertility; gestational week at birth; birth weight of the infants; 5-minute APGAR score; and serum levels of folate, vitamin B12, and homocysteine

Variables	Median	Minimum-Maximum
Maternal Age (years)	30	21-41
Number of previous IVF cycles	2	1-6
Duration of infertility (months)	24	12-72
Gestational week at birth	37	34-39
Birth weight of the infants (g)	3030	2100-3780
Fifth minute APGAR Score	10	7-10
Serum folate levels (nmol/L)	16.3	5.7-27
Serum vitamin B12 levels (ng/L)	289	129-465
Serum homocysteine levels (µmol/L)	8.1	3.4-12.4

Table 2 shows the proportions for autoantibodies. Table 3 shows the proportions for hereditary thrombophilias, MTHFR polymorphisms, and autoantibody positivity together with thrombophilias.

**Table 2:** Percentage of autoantibodies

Autoantibody	Percentages
anti-TPO	17/53 (32.1%)
anti-TG	11/53 (20.7%)
ANA	8/53 (15.1%)
Anti-dsDNA	4/53 (7.5%)
ASMA	1/53 (1.9%)
aCL IgG-IgM	1/53 (1.9%)

anti-TPO: anti- thyroid peroxidase, anti-TG: antithyroglobulin, ANA: antinuclear antibodies, anti-dsDNA: anti-double stranded DNA antibody, ASMA: anti-smooth muscle antibody, aCL: anticardiolipin antibody.

**Table 3:** Proportion of hereditary thrombophilias, MTHFR polymorphisms, and autoantibody positivity together with thrombophilias

Variables	Proportion
MTHFR C677T Heterozygous	29/53 (54.7%)
MTHFR C677T Homozygous	9/53 (17.0%)
MTHFR A1298C Heterozygous	28/53 (52.8%)
MTHFR A1298C Homozygous	8/53 (15.1%)
MTHFR Compound Heterozygous	21/53 (39.6%)
F 5 Leiden Heterozygous	10/53 (18.9%)
F 5 Leiden Homozygous	2/53 (3.8%)
Prothrombin 20210 A Heterozygous	2/53 (3.8%)
Prothrombin 20210 A Homozygous	0/53 (0.0%)
PAI-1 4G/5G Heterozygous	11/53 (20.7%)
PAI-1 4G/5G homozygous	2/53 (3.8%)
Autoantibody positivity with thrombophilia	28/53 (52.8%)

MTHFR: Methylene tetrahydrofolate reductase, PAI: Plasminogen activator inhibitor

Twenty-three patients (43.4%) had Hashimoto's thyroiditis; seven (13.2%) had SLE; one (1.9%) had Behcet's Disease; one had Sjogren's syndrome; one (1.9%) had ulcerative colitis; and one (1.9%) had anti-phospholipid antibody syndrome.

The 53 patients had at least one hereditary thrombophilia and/or MTHFR polymorphism. Autoantibody positivity was seen in 28 patients (52.8%) combined with hereditary thrombophilia and/or MTHFR polymorphisms.

## DISCUSSION

Although IVF is the most successful treatment method for UI, its overall live birth rate is approximately 40%, depending on maternal age and the number of transferred embryos (Elizur *et al*., 2006). Infertility is a multifactorial condition that affects couples in medical, psychological, sociological, and economic aspects (Péloquin *et al*., 2018). Some identifiable risk factors are genetic abnormalities, ovulatory disorders, tubal damage, uterine or peritoneal problems, and male factors (Ray *et al*., 2012). On the other hand, nearly 30% of couples have no determined etiology (Ray *et al*., 2012).

The complexity of the situation emphasizes the need for further investigation. Hyperhomocysteinemia, MTHFR gene polymorphisms, variations in folate pathway genes, vitamin B-complex deficiencies, hereditary thrombophilias, and autoimmune antibody production are some factors that contribute to the etiology of UI (Deroux *et al*., 2017; Altmäe *et al*., 2010; Das *et al*., 2015; Hou *et al*., 2016; Enciso *et al*., 2016).

Thrombophilia can cause infertility as it impairs oocyte quality, increases hypoxic damage, and diminishes vascularization (Qublan *et al*., 2006). Properly vascularized and oxygenated follicles lead to higher rates of implantation (Qublan *et al*., 2006; Chui *et al*., 1997). It has been shown that during IVF the mean follicular diameter, oocyte retrieval rate, number of mature oocytes, and fertilization rates are higher in highly vascularized follicles (Qublan *et al*., 2006; Bhal *et al*., 1999). Implantation, placentation, angiogenesis, and vascular remodeling require an appropriate balance between coagulation and fibrinolysis, although thrombosis in the intervillous space and placental vessels may cause placental insufficiency (Qublan *et al* ., 2006). On the other hand, heparin has a role in trophoblast differentiation and invasion (Lodigiani *et al*., 2017; Nelson & Greer, 2008). More specifically, low-molecular-weight heparin (LMWH) affects matrix metalloproteinases (MMP), tissue inhibitors, cadherin-E, heparin-binding epidermal growth factor (HBEGF), and insulin-like growth factor (IGF) (Lodigiani *et al*., 2017; Nelson & Greer, 2008; Di Simone *et al*., 2007; Erden *et al*., 2006; Das *et al*., 1994; Lacey *et al.*, 2002). LMWH also has a positive effect on IVF outcomes (Lodigiani *et al*., 2017). These findings support our results, which indicated that appropriate management of thrombophilia and proper therapy with heparin play a significant role in the treatment of UI.

Methionine is an essential amino acid in nucleic acid synthesis and homocysteine is a metabolite resulting from its metabolism (Das *et al*., 2015). High levels of homocysteine may arise from deficiency of certain vitamins (folic acid, vitamin B6, etc.) and/or MTHFR gene polymorphisms (Das *et al*., 2015). Hyperhomocysteinemia causes many adverse pregnancy outcomes, including pregnancy loss, neural tube defects, chromosomal aneuploidies, fetal cardiac defects, preeclampsia, placental abruption, and intrauterine growth restriction (IUGR) (Das *et al*., 2015; Ray & Laskin, 1999; Botto & Yang, 2000). Hyperhomocysteinemia has also been associated with impaired follicular development, oxidative damage to oocytes, improper vascularization of the chorionic villi, and implantation failure (Das *et al*., 2015; Jerzak *et al*., 2003). Therefore, deficiencies in folate and/or B-complex vitamins lead to the accumulation of homocysteine and may cause UI (Altmäe *et al*., 2010). Some of the hypothesized mechanisms include impaired cell division, increased production rates of inflammatory cytokines, impaired nitric oxide metabolism, increased oxidative stress, increased rates of apoptosis, and impaired methylation reactions. All these factors combined affect oocyte development and embryo implantation and reduce endometrial receptivity (Altmäe *et al*., 2010). Thus, supplementation with folate and B-complex vitamins before conception decreases homocysteine levels in the follicular fluid, which leads to decreased rates of infertility and lower rates of miscarriage (Altmäe *et al*., 2010; Chavarro *et al*., 2008). Heterozygous mutations of the gene coding the MTHFR enzyme decrease enzyme activity by 35%, while homozygous mutations decrease activity by 70%, thus causing impaired folate metabolism and accumulation of homocysteine (Frosst *et al*., 1995). Several studies in the literature have described the negative effects of high homocysteine levels in patients with infertility and/or polycystic ovary syndrome (PCOS) (D'Uva *et al*., 2007; Bibi *et al*., 2010; Schachter *et al*., 2003). These findings are consistent with our results. Appropriate management of decreased serum homocysteine levels and increasing serum folate and vitamin B levels are essential for the treatment of UI.

Autoimmune factors seem to play a pivotal role in infertility (Chen *et al*., 2017). The prevalence of antiphospholipid antibodies (aPL), ANA, and antithyroid antibodies was found to be particularly higher in women with UI (Chen *et al*., 2017). Implantation is a process that necessitates immunologic tolerance and requires cross-talk between the embryo and the maternal immune system (Kushnir *et al*., 2016). Impaired implantation and high pregnancy loss rates in women with autoimmune antibodies may be caused by injury to the syncytiotrophoblasts, endovascular trophoblasts covering the tip of the spiral arteries, endothelial cells of the spiral veins, superficial/glandular epithelial cells of the decidua, autoantibody inflammatory processes, and entrance of cell degradants from these tissues into the maternal circulation. These biological events result in impaired implantation and disturbed fetal perfusion (Beksaç *et al*., 2017). In addition to these, ovulation, implantation, and onset of labor are thought to be associated with inflammation (Kushnir *et al*., 2016; Weiss *et al*., 2009). Although exact screening and management protocols for infertile patients positive for autoimmune antibodies have not been established, it seems reasonable to devise a patient-centered, individualized approach for these patients. Low-dose methylprednisolone (4 mg, once a day), low-dose acetylsalicylic acid (100 mg once a day), and low-dose LMWH (20 mg/0.2 ml enoxaparin sodium administered intramuscularly once a day) were the main components of treatment, as it has been shown that heparin and heparin-related derivatives have an anti-inflammatory effect (Young, 2008).

Although IVF is a revolutionary treatment for infertile couples, use without appropriate indication may increase both costs and procedure-related complications (Ben-Ami *et al*., 2017). Ovarian hyperstimulation syndrome, multiple pregnancy, preterm labor, preeclampsia, and increased rates of chromosomal/structural abnormalities are some of the complications related with the IVF procedure (Ben-Ami *et al*., 2017; Xu *et al*., 2015).

The 53 patients had at least one underlying condition (hereditary thrombophilia, MTHFR gene polymorphism, or autoantibody positivity). After the introduction of appropriate management protocols, all patients delivered healthy babies. Preeclampsia, eclampsia, and placental abruption were not observed in any of the patients. There were only three spontaneous late preterm deliveries (5.6%) (at 34, 35 and 35 weeks of pregnancy, respectively).

The limitations of this study are the small number of patients, its retrospective design, and the fact that it reflects the experience of a single center. Furthermore, this is a descriptive assessment of a very specific group of patients who gave birth at our institution without the aid of assisted reproductive technology and who had a history of failed IVF cycles for UI. This study was not designed to investigate the success rate of our management protocol, but to stress the importance of knowing the etiological factors behind UI.

In conclusion, infertility is a field with plenty to discover. Underlying maternal conditions may cause conception failure. Evaluation and management of hereditary thrombophilias, MTHFR gene polymorphisms, and/or autoimmune conditions may be beneficial for primary infertility patients with at least one failed IVF cycle.
